# Effect of epidural spinal cord stimulation in individuals with sensorimotor complete spinal cord injury: a pilot study

**DOI:** 10.3389/fnsys.2025.1622033

**Published:** 2025-07-02

**Authors:** Vojtech Rybka, Kristyna Sediva, Lenka Spackova, Pavel Kolar, Ondrej Bradac, Jiri Kriz

**Affiliations:** ^1^Spinal Cord Unit, Department of Rehabilitation and Sports Medicine, Second Faculty of Medicine, Charles University and University Hospital Motol, Prague, Czechia; ^2^Department of Rehabilitation and Sports Medicine, Second Faculty of Medicine, Charles University and University Hospital Motol, Prague, Czechia; ^3^Department of Neurosurgery for Children and Adults, Second Faculty of Medicine, Charles University and University Hospital Motol, Prague, Czechia

**Keywords:** epidural spinal cord stimulation, spinal cord injury, functional improvement, weight-bearing standing, autonomic dysfunction

## Abstract

Spinal cord injury (SCI) often results in irreversible sensorimotor and autonomic dysfunction, severely limiting independence and quality of life. Individuals with clinically complete SCI (AIS A) have traditionally been considered beyond functional recovery. This pilot study aimed to evaluate the therapeutic potential and broader impact of epidural spinal cord stimulation (eSCS) on this population. Three participants with chronic, sensorimotor complete thoracic SCI received implantation of a 32-contact epidural stimulator targeting the lumbosacral spinal cord. Personalized stimulation protocols were developed to support lower limb activation and upright posture. Motor, autonomic, and quality of life outcomes were assessed over a 12-month follow-up. All participants achieved independent standing through eSCS-enabled muscle activation, despite the absence of voluntary movement. The intervention led to improvements in postural control and trunk stability, as well as clinically meaningful gains in autonomic functions, including bladder, bowel, and sexual health, alongside reductions in spasticity and neuropathic pain. Importantly, participants reported sustained and substantial improvements in their perceived quality of life. This study provides evidence that eSCS can restore key functional capacities and significantly enhance the quality of life for individuals with sensorimotor complete SCI.

## 1 Introduction

Spinal cord injury (SCI) leads to permanent impairment of sensorimotor and autonomic functions. The neurological deficit primarily results in the loss of voluntary movement, leading to the inability to stand or walk. However, dysfunction of the autonomic nervous system (ANS) often presents as a significant obstacle, such as bladder and bowel emptying disorders or sexual dysfunction.

In recent decades, there has been an increasing number of studies providing evidence of the positive effects of epidural spinal cord stimulation (eSCS) in individuals with chronic complete SCI. Stimulation of the lumbosacral spinal cord activates the muscles of the lower extremities and can partially restore standing and walking (Angeli et al., 2018; Wagner et al., 2018). Furthermore, recent research has shown that eSCS can also restore voluntary movement below the level of a clinically complete SCI (Peña Pino et al., 2020). Simultaneously, the effects of eSCS on the ANS are being monitored (Darrow et al., 2019). Several authors have confirmed the positive effects of stimulation on cardiovascular function (Bloom et al., 2020; Legg Ditterline et al., 2020) as well as neurogenic bladder and bowel (Herrity et al., 2018; Walter et al., 2018).

Our study focuses on monitoring the effects of eSCS on sensorimotor and autonomic functions in chronic patients with clinically complete (AIS A) thoracic SCI (NCT05690074). We hypothesized that the activation of muscle groups in the lower limbs would be beneficial for verticalization. We also hoped for partial restoration of functional connectivity at the level of lesion. Additionally, we anticipated changes in spasticity and neuropathic pain. Given the rare reports of a positive effect of eSCS on autonomic functions, we aimed to verify the improvement in cardiovascular response, micturition, defecation, and sexual function in our patients.

## 2 Materials and methods

### 2.1 Participants

Three participants were included in the study based on the following criteria: sensorimotor complete traumatic SCI (AIS A) at the upper thoracic segments T1–T6; neurologically stable for at least 1 year post-injury; at least 18 years old; without medical complications (intractable pain, non-healing fractures, contractures, pressure ulcers, urinary infections, or comorbidities); stable medication; and a stable psychological condition, without abuse.

All three participants were male, with at least 2 years since their injury, and had neurological lesions at the T2–T4 level. The mean age was 29.8 (±3.4) years at the time of implantation (Table 1). Somatosensory evoked potentials (SSEP) for the upper and lower limbs, along with functional magnetic resonance imaging (fMRI) of the brain, were conducted before implantation to map the cerebral response to the intended movement of the upper and lower limbs. All participants exhibited normal SSEP from the upper limbs and no response from the lower limbs. Similarly, fMRI showed no cerebral response for the lower limbs. These examinations confirmed no functional connectivity between supraspinal and spinal centers located below the level of the spinal cord lesion.

**TABLE 1:** Characteristics of study participants.

Participant	Age (years)	Gender	Cause	Post injury (years)	NLI	AIS	Sensory subscores				SSEP
							LTR	LTL	PPR	PPL	TN
A	32.0	Male	Bike	3.1	T2	A	19	18	18	20	0
B	25.0	Male	Motorbike	2.4	T4	A	23	23	22	22	0
C	32.3	Male	Motorbike	4.2	T4	A	22	23	22	22	0

NLI, neurological level of injury; AIS, ASIA Impairment Scale; SSEP, somatosensory evoked potentials; LTR, light touch right; LTL, light touch left; PPR, pin prick right; PPL, pin prick left; TN, tibial nerve.

All participants signed informed consent for the study, which was approved by the ethics committee of Motol University Hospital (EK-1302/21). Two participants (A and B) were subjects of a previous case study (Rybka et al., 2024), which evaluated the effect of stimulation on improving sexual function.

None of the participants received any standing or walking training prior to the placement of the neurostimulator.

### 2.2 Implantation of the neurostimulator

Following preoperative tests, all participants underwent the implantation of a spinal cord stimulator with a 32-contact electrode (WaveWriter Alpha IPG, CoverEdge X32, Boston Scientific, USA). The stimulating electrode was placed in the conus medullaris area to target the L1-S1 segments (Mesbah et al., 2021). Following verification of the conus medullaris topography using MRI, the electrode was inserted into the dorsal epidural space under fluoroscopic control. Evoked motor responses from needle electrodes inserted into the iliopsoas, vastus lateralis, biceps femoris, tibialis anterior, medial gastrocnemius, and soleus muscles were utilized to determine the precise position of the stimulating electrode. The pulse generator was implanted subcutaneously in the left mesogastrium.

### 2.3 Electrode configuration

One week after the surgical implantation of the neurostimulator, spatiotemporal mapping of muscle responses in the lower limbs was initiated. In the supine position, stimulation was set to a frequency of 20 Hz for the activation of extensor groups and 100 Hz for the activation of flexor groups (Rowald et al., 2022), with a pulse width of 210–400 ms and an intensity of 2–15 mA. The contacts on the electrode were successively switched rostrocaudally for the right and left sides, and motor responses were monitored visually, palpated, and electromyographically. EMG signals were recorded using surface electrodes from the iliopsoas, rectus femoris, vastus lateralis, biceps femoris, tibialis anterior, medial gastrocnemius, soleus, and gluteus maximus muscles. The stimulation parameters were optimized to achieve the most isolated activation of individual muscles for targeted movement in a given joint.

In addition to the supine position, the lateral lying position with the lower limb suspended in the Redcord system was utilized for flexion and extension training. In the sitting position, a lower limb extension program for the knee joint was implemented. For standing training, standard programs for bilateral extension of the knee joints were employed, either alone or in combination with activation of the gluteal muscles. Due to the coactivation of the ischiocrural muscles interfering with knee joint extension, the pulse frequency was reduced to 10 Hz, which facilitated more targeted activation of the quadriceps femoris muscle. Despite the pronounced rhythmic activity, improved quality and longer duration of standing were achieved. A subthreshold stimulation program using caudal electrode contacts was established to potentially influence autonomic functions.

### 2.4 Training program

During a 2-week hospitalization, the configuration of the electrodes was completed, and the first verticalization to standing using a forearm walker was performed. Support was provided for the knee joints during the standing and transition phases to prevent sagging. Assistance to maintain the correct position of the pelvis was necessary only when changing positions from sitting to standing and back. After short-term testing, the initially considered body weight support system proved ineffective due to the patient’s discomfort and the need for more therapists to provide assistance.

After discharge, home stimulation continued in lying and sitting positions for 2–3 h a day, divided into at least two exercise sessions. Each session included individualized programs designed to stimulate each limb and muscle group separately, allowing participants to focus entirely on the intended movements. This approach aimed to promote the most effective activation of the spinal cord pathways. The knee extension program was conducted in a sitting position, while hip flexion and knee extension exercises were performed in a lying position. Stimulation programs for each muscle group alternated every 5 min or when the muscle’s motor activity was exhausted. Additionally, each exercise session included a program to influence the ANS, which ran continuously for 20–30 min.

Participants attended weekly outpatient check-ups, during which stimulation parameters were adjusted, and if necessary, muscle responses were remapped using visual and palpation assessments of activity. The goal was to maintain optimal motor responses in the stimulated muscle groups while minimizing coactivation of other muscles. Independent standing in a walker could begin after achieving adequate muscle strength in the lower limbs. This was defined as the ability to maintain knee extension with the stimulation turned on while sitting for at least 10 s. If participants could stand without assistance in a forearm walker, they could include standing training in home therapy and start training verticalization in a standard (low) walker. Participants continued to use the stimulation programs in supine and sitting positions. For standing in a walker, it was necessary to adjust the stimulation parameters, especially intensity and frequency, to optimally utilize the supportive function of the lower limbs.

### 2.5 Examination

Changes in sensorimotor functions were assessed according to the International Standards for Neurological Classification of Spinal Cord Injury (ISNCSCI). The Trunk Stability Test and the Locomotor Stages in Spinal Cord Injury (LOSSCI) (Maurer-Burkhard et al., 2016) were used to objectify postural functions. Respiratory muscle strength was monitored using spirometry. fMRI and SSEPs assessed the potential restoration of functional connections between supraspinal and spinal centers below the level of lesion. During the fMRI examination, brain activity was evaluated at rest and during active or intended movements of the patient’s upper and lower limbs, following instructions on the screen (Supplementary Method). SSEPs were recorded according to a standard protocol in response to median nerve stimulation at the wrist and the tibialis nerve dorsal to the medial malleolus. Spirometry was performed using the Pony FX device, monitoring dynamic parameters FVC, FEV1, PEF, and indicators of respiratory mechanics (MIP/MEP).

Spasticity was assessed using the Modified Ashworth Scale (MAS) and Muscle Excitability Scale (MES) (Kriz et al., 2024), and the Spinal Cord Injury Spasticity Evaluation Tool (SCI-SET) questionnaire. Neuropathic pain was recorded using the Neuropathic Pain Scale (NPS), and quality of life was evaluated using the WHOQOL-BREF questionnaire.

The Head-Up Tilt Test (HUTT) was used to examine cardiovascular instability. It was performed as a standard procedure on a verticalization table tilted up to 60°, while continuous blood pressure (BP) monitoring was conducted with a finger cuff using the Finapres Nova device (Finapres Medical Systems, Netherlands). Symptoms were recorded using the Autonomic Dysfunction Following Spinal Cord Injury (ADFSCI) questionnaire.

Urodynamic examination assessed lower urinary tract dysfunction. The examination was performed according to the recommendations of the International Continence Society (Rosier et al., 2017). Long-term treatment with parasympatholytics was continued. Filling cystometry was conducted in the supine position with continuous filling of sterile water at 32–35°C at a rate of 50 ml/min. Water volume, bladder pressure, and uninhibited contractions were recorded. The examination was terminated when a volume of 500 ml was reached, spontaneous urine leakage occurred, intravesical pressure exceeded 40 mmH_2_O, or autonomic dysreflexia developed. Additionally, the Neurogenic Bladder Symptom Score (NBSS), Neurogenic Bowel Dysfunction Score (NBDS), and International Index of Erectile Function (IIEF-5) questionnaires were used.

All questionnaire assessments, spirometry, trunk stability tests, and spasticity assessments were performed at 0, 3, 6, 9, and 12 months, while ISNCSCI, fMRI, SSEP, and UD were conducted at 0, 6, and 12 months.

## 3 Results

### 3.1 Effect of eSCS on sensorimotor functions

**FIGURE 1:**
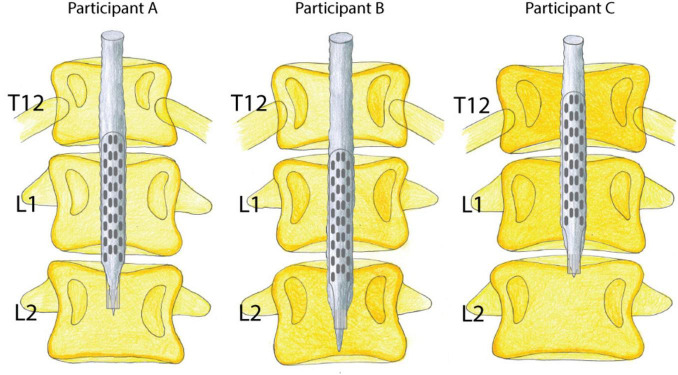
Visualization of paddle electrode placement in relation to the conus medullaris and vertebral bodies. Lumbosacral spinal cord reconstruction from MRI scans and X-rays; estimated epidural stimulation paddle placement in all three participants.

**FIGURE 2:**
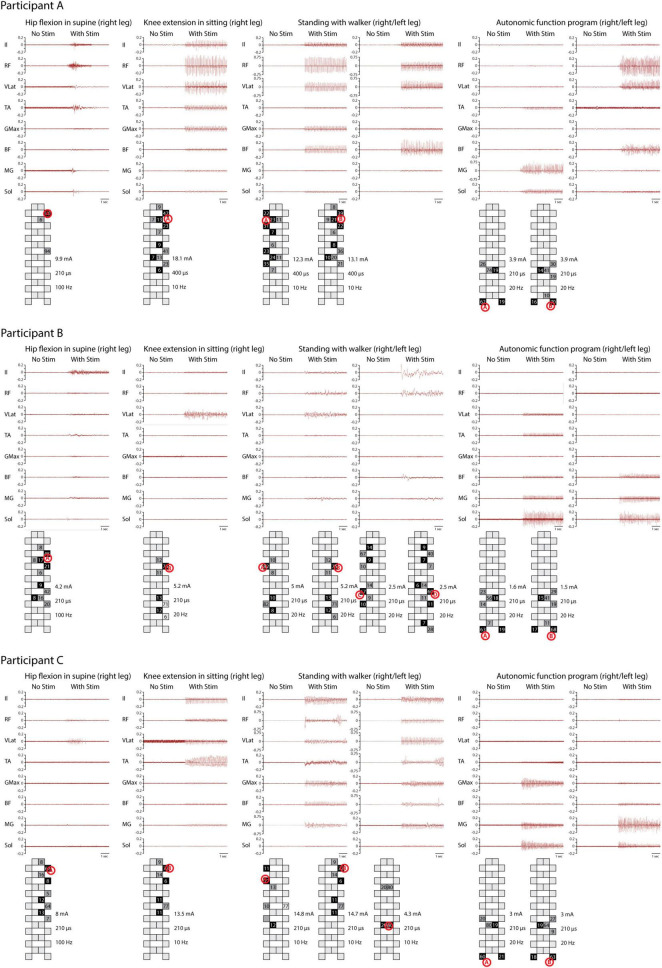
Electromyography (EMG) activity during four training programs. EMG activity was recorded from the iliopsoas (Il), rectus femoris (RF), vastus lateralis (VLat), tibialis anterior (TA), gluteus maximus (GMax), biceps femoris (BF), medial gastrocnemius (MG), and soleus (Sol) during hip flexion in supine, knee extension in sitting, and full weight-bearing standing, as well as a program for potential modulation of autonomic functions. Different patterns of muscle activity are evident based on electrode configuration. Stimulation intensity, frequency, pulse width, and electrode configuration are documented. The system used calculates the distribution of cathodes (black) and anodes (gray) according to the stimulated site (red letter in circle). For standing, a different number of programs were applied for each subject. EMG activity is recorded solely during stimulation.

All three participants with sensorimotor complete SCI generated motor responses in both lower limbs during individually adjusted stimulation programs for both supine and sitting positions. Electrode configurations differed slightly in their placement on the lumbosacral enlargement of the spinal cord (Figure 1). Motor responses were recorded electromyographically and occurred only during stimulation (Figure 2). However, none of the participants could generate EMG activity or perform limb movements in response to verbal commands without an active stimulation program.

#### 3.1.1 Restoration of the ability to stand

**FIGURE 3:**
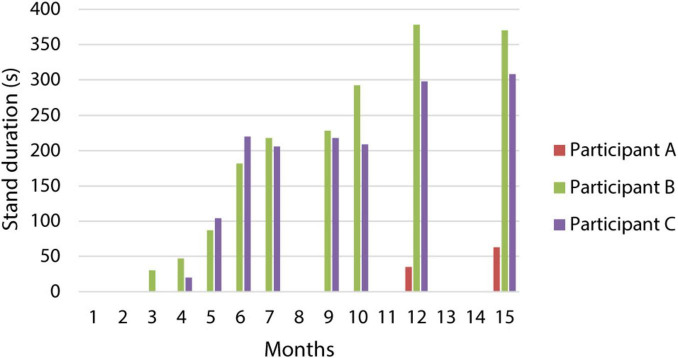
Changes in the duration of standing with a standard walker. The graph describes the increase in stand duration over a 15-month period for all three participants.

Appropriate activation of the extensors during the standing stimulation program initially enabled standing in a forearm walker with significant support from the upper extremities, particularly during the transition from sitting to standing. Gradually, all participants achieved independent standing in a standard walker with a high-quality postural pattern, without assistance (Figure 3 and Supplementary Video 1). Participants B and C were able to stand in a standard walker by the third and 4th months after implantation, respectively, with a gradual increase in standing duration. By the 9th month, both could perform short-term differentiation of the lower limbs’ activity using a modified program that allowed standing on one leg. They were also able to manage stimulation in more challenging developmental positions. For instance, the stimulation program designed for standing in a high kneeling position facilitated better alignment of the pelvis and lumbar spine. Participant A was able to stand independently in a standard walker up to 11 months after implantation, despite facing complicated stimulation settings and internal complications.

#### 3.1.2 Changes in neurological condition

The evaluation at 6 and 12 months revealed trends in objective and subjective indicators. First, no significant changes in the neurological condition were observed. At 1 year post-implantation, the ISNCSCI examination showed persistent sensorimotor completeness (AIS A) in all three participants. The neurological level of the lesion decreased only in participant A, from T2 to T3. In participants A and B, an average increase of 1.3 points in the sensory score was recorded (Supplementary Table 1). SSEPs with the stimulation turned off exhibited no response to stimulation of the tibialis nerve in participants A and B. In participant C, a minimal response of up to 1.5 μV was observed. Functional MRI with a motor paradigm focused on the intended movement of the lower limb’s fingers, performed at the same time interval, did not show significant changes in the activation maps in the relevant motor area for any of the three participants.

#### 3.1.3 Changes in stability

All participants reported subjective improvements in sitting stability during everyday activities, such as transfers and wheelchair use, a year after the start of the study, with stimulation turned off. However, these changes were not reflected in the trunk stability test; only participant B showed an improvement in the subscore for dynamic activity involving upper limbs. The same participant showed an improvement in the LOSSCI, moving from stage III (creeping) to IVa (functional climbing with three support points).

#### 3.1.4 Changes of spasticity and neuropathic pain

**FIGURE 4:**
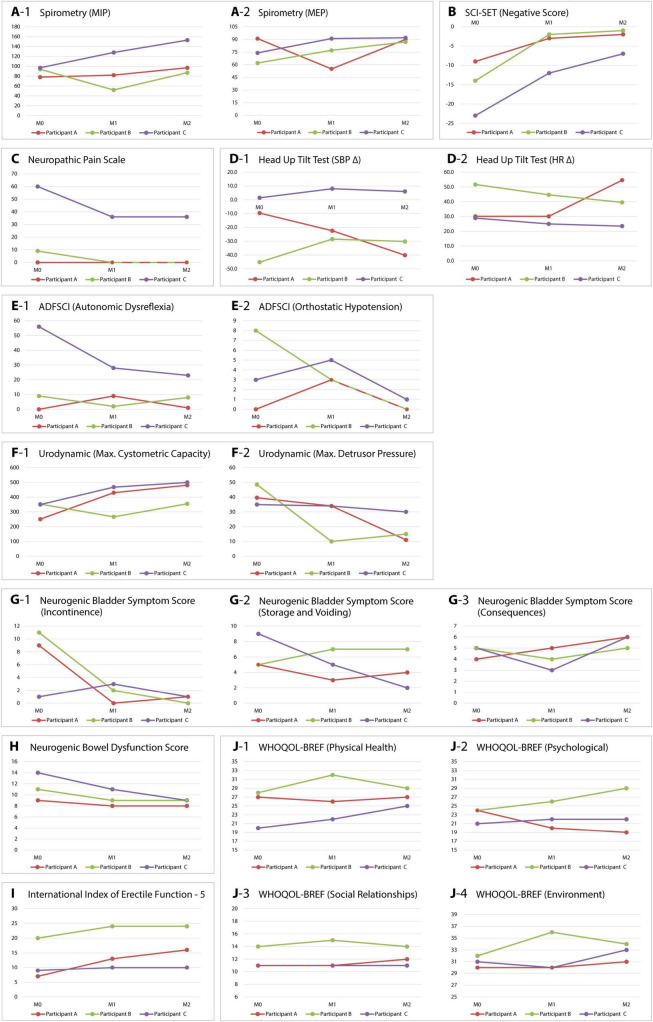
Changes in monitored parameters of sensorimotor and autonomic functions. Variables were recorded before implantation (M0), at 6 months (M1), and after 1 year (M2). The graphs illustrate changes in respiratory functions **(A-1,A-2)**, spasticity **(B)**, neuropathic pain **(C)**, cardiovascular functions **(D-1,D-2,E-1,E-2)**, urology **(F-1,F-2,G-1–G-3)**, bowel function **(H)**, sexual function **(I)**, and quality of life **(J-1–J-4)** for all three participants. MIP, maximum inspiratory pressure; MEP, maximum expiratory pressure; SCI-SET, spinal cord injury spasticity evaluation tool; SBP Δ, difference in systolic blood pressure between 0 and 60° tilt; HR Δ, difference in heart rate between 0 and 60° tilt; ADFSCI, autonomic dysfunction following spinal cord injury; WHOQOL-BREF, World Health Organization quality of life – abbreviated version.

Spasticity was mainly reduced in the subjective assessment. In the SCI-SET questionnaire, the negative score was significantly reduced in all participants, averaging 12 points (Figure 4B). The differences in MES and MAS did not show a clear trend. Neuropathic pain, present only in participant C, was reduced in the NPS from 60 to 36 points (Figure 4C).

#### 3.1.5 Changes in respiratory parameters

Spirometry indicated non-significant changes in the FVC and FEV1 parameters. However, participants B and C demonstrated a significant improvement in the maximum expiratory pressure (MEP). In participant A, this parameter remained unchanged. Additionally, it was significantly reduced at 6 months, likely due to ileus resulting in subsequent restriction of the regimen (Figure 4A).

### 3.2 Effect of eSCS on autonomic functions

#### 3.2.1 Changes in cardiovascular functions

The differences in systolic blood pressure (SBP) and heart rate (HR) between the supine and 60° positions did not reveal a clear trend. Only participant B displayed a notable reduction in orthostatic hypotension during verticalization (Figure 4D). Before initiation of the study, subjective symptoms of autonomic dysreflexia and orthostatic hypotension were recorded in participants B and C using the ADFSCI questionnaire. In the year following the implantation, participant B experienced a reduction of 8 points in OH symptoms, while participant C demonstrated a decrease of 33 points in AD symptoms. Other changes were insignificant (Figure 4E).

#### 3.2.2 Changes in micturition, defecation, and sexual functions

All participants had clinically significant lower urinary tract and bowel dysfunction before the study. Urodynamic examination, namely filling cystometry, showed differences during the observed period. In all three participants with stable parasympatholytic medication, there was an increase in maximum bladder capacity and a decrease in maximum detrusor pressure compared to the examination before the stimulator was inserted (Figure 4F). The NBSS questionnaire indicated a significant reduction in symptoms related to neurogenic bladder in all three participants during the year following the implantation of the stimulator. The most significant changes were noted in participants A and B, particularly in the incontinence domain (8 and 11 points, respectively) (Figure 4G). Bowel dysfunction, assessed by the NBDS, also improved in all three participants, with participants B and C moving from moderate to mild in the NBDS severity assessment (Figure 4H). Sexual dysfunction, evaluated using the IIEF-5 questionnaire, also improved in all participants, with the most significant changes observed in participant A (9 points) (Figure 4I).

### 3.3 Effect of eSCS on quality of life

We also monitored the impact of eSCS on the quality of life of our participants. Using the WHOQOL-BREF tool, we assessed possible changes in four domains: physical health, experience, social relationships, and environment. The results were inconsistent; only in the environment domain was a positive trend indicated for all participants (Figure 4J).

## 4 Discussion

### 4.1 Effect of eSCS on sensorimotor functions

The results of our study confirm the effect of eSCS on the controlled activation of lower limb muscles in individuals with sensorimotor complete SCI. They are consistent with the conclusions of other studies that also addressed the possibility of partial recovery of motor functions without supraspinal control (Angeli et al., 2018; Wagner et al., 2018). Despite adequate motor activation during stimulation, we were unable to demonstrate recovery of voluntary movement. We believe that participant selection is a crucial factor. Our aim was to investigate the use of eSCS solely in patients with sensorimotor complete lesions per the ISNCSCI. None of the participants exhibited any clinical or electrophysiological changes that would suggest incomplete lesions, as described by other authors (Angeli et al., 2014; Rejc et al., 2020). We did not observe changes indicating possible restoration of functional connectivity with supraspinal centers in either SSEPs or fMRI. Although some studies describe potential plastic changes in spinal circuits, direct evidence of the restoration of corticospinal communication is still lacking (Eisdorfer et al., 2020).

The highest postural position achieved in our study using eSCS is independent standing in a low walker or standing on one leg. The key to this function was targeted stimulation focused on extension in the knee and hip joints and optimal adjustments regarding the loading of joint structures. The potential of using eSCS for targeted muscle activation with a functional impact aligns with other works (Mesbah et al., 2021). It emphasizes the need for careful adjustment of frequency and amplitude, which are necessary for selecting the desired muscle groups (Rejc et al., 2017). Unlike other studies (Wagner et al., 2018; Rowald et al., 2022), we did not continue to train the walking mechanism after achieving full weight-bearing standing. The reason was the technical limitations of our system, which do not allow for a smooth transition between individual stimulation programs.

An important aspect of our work was the subjective assessment of the study participants, which indicated an improvement in functional trunk stabilization applicable to activities of daily living. Unfortunately, this does not correlate with the results of objective tests (e.g., the Trunk Stability Test), which did not demonstrate this effect. However, we noted a significant improvement in the respiratory parameter MEP in participants B and C, which may be attributed to the enhancement in the function of the trunk expiratory muscles. This connection was demonstrated, for instance, by the study by Roth et al. (2010). Participant A exhibited limiting respiratory insufficiency during standing. In this position, there was an anteversion of the pelvis and accentuated lordosis in the lumbar spine. The study by Hwang and Kim (2018) demonstrated that pelvic anteversion can restrict diaphragm movement and decrease vital lung capacity due to increased chest rigidity and suboptimal adjustment of the respiratory muscles. The participant’s respiratory discomfort was alleviated by changing the position of the pelvis from anteverted to neutral. It was initially managed by manual assistance; however, after utilizing eSCS as part of physiotherapy in various postural positions, we successfully corrected the inappropriate pelvic position and respiratory discomfort.

In our study, we also investigated the effect of eSCS on spasticity. In contrast to the inconclusive changes in the clinical assessment of spasticity, the subjective assessment of all three participants using the SCI-SET tool showed a significant reduction in the negative impact of spasticity, particularly on ADL. This trend aligns with the conclusions of reviews confirming that eSCS can contribute to reducing spasticity in individuals with SCI (Jung et al., 2024; Hachmann et al., 2021). We also observed a similar effect of eSCS on neuropathic pain in participant C.

### 4.2 Effect of eSCS on autonomic functions

A secondary objective of our study was to investigate the effect of eSCS on the ANS. Only participant B reported symptoms of orthostatic hypotension before eSCS. After a year of regular stimulation, his symptoms improved, which was also reflected in the reduction of the hypotensive response during HUTT. However, BP was not completely stabilized. Studies investigating the effect of eSCS on orthostatic hypotension have noted stabilization of BP during verticalization with stimulation on (West et al., 2018; Aslan et al., 2018). In contrast, we intentionally performed HUTT with stimulation off to assess the effect of long-term SCS on potential changes in sympathetic nervous system reactivity during orthostatic stress.

We noted positive results in evaluating the effect of eSCS on neurogenic bladder and bowel dysfunction. Filling cystometry demonstrated improved bladder capacity and decreased detrusor pressure, accompanied by the disappearance of incontinence episodes in participants A and B. However, no participant restored the ability to void voluntarily. It should be reiterated that the aim was to investigate the long-term effects of SCS on autonomic functions, not the direct impact on the initiation of micturition, as examined by Herrity et al. (2022). All our patients also reported reduced bowel dysfunction symptoms as captured by the NBDS questionnaire. The positive effect of eSCS on neurogenic bladder and bowel dysfunction was also noted by Kandhari et al. (2022). In agreement with these authors, we noted a reduction in erectile dysfunction in all three participants using the IIEF-5 questionnaire. Additionally, participants A and B managed to improve the ejaculatory reflex, as we presented in a previous communication (Rybka et al., 2024).

### 4.3 Effect of eSCS on quality of life

We expected that the effect of SCS on motor and autonomic functions would also be reflected in the assessment of quality of life using the WHOQOL-BREF tool. Although all participants unanimously reported a positive effect of SCS on various aspects of quality of life, the questionnaire did not fundamentally reflect these changes. The question arises whether the optimal assessment method was chosen. The authors Boakye et al. (2023) confirmed improvements in various domains of quality of life when assessing the effect of SCS in 25 patients using a structured interview. The authors Huang et al. (2022) conducted a literature review regarding the benefits of SCS on the quality of life of patients after SCI. They confirmed the benefit of SCS for pain relief, spasticity, improvement of motor functions, and defecation. However, these studies did not directly assess quality of life with a specific tool.

### 4.4 Complications of eSCS

Although eSCS is generally considered a safe method, it does carry certain risks related to both the surgical procedure and the hardware used. Intraoperative complications can include wound infections, hematomas at the generator site, or cerebrospinal fluid leakage. In the subsequent phase, issues such as electrode migration or stimulator failure may arise (Verrills et al., 2016). Additionally, using eSCS in patients with SCI can lead to specific adverse effects, including muscle loss, osteoporosis, spasticity, or the risk of autonomic dysreflexia (Boakye et al., 2023).

During the initiation of verticalization in a forearm walker, we observed respiratory discomfort in participant A. This issue was alleviated by manually adjusting the pelvis from anteversion to a neutral position. Additionally, the participant developed ileus 6 months after implantation, which was resolved conservatively, although it required load limitation for 6 weeks. As a result, participant A was able to stand independently in a standard walker only up to 11 months after implantation. Participant C developed an incipient pressure ulcer at the lower edge of the stimulator after 11 months due to migration within the subcutaneous tissue. This caused irritation of the left abdominal wall during stimulation while in the standing position. After undergoing surgical revision and repositioning of the stimulator under the fascia, the issues were resolved.

## 5 Conclusion

Our pilot study confirmed that eSCS can facilitate targeted activation of lower limb muscles and allow individuals with complete sensorimotor SCI to stand independently. Although we could not restore voluntary movement or demonstrate signs of reconnection between supraspinal and spinal centers, we observed several significant improvements in autonomic functions, trunk stability, and the subjective perception of quality of life. We regard it as a noteworthy benefit that, in addition to enabling independent standing, the other improvements achieved do not rely on active stimulation.

The current configuration of the employed stimulator does not yet allow for a sequence of programs tailored to the gait cycle. However, the results indicate significant therapeutic potential for eSCS in promoting functional independence and improving the quality of life for this specific group of patients.

## Data Availability

The raw data supporting the conclusions of this article will be made available by the authors, without undue reservation.

## References

[B1] Angeli C. A. Boakye M. Morton R. A. Vogt J. Benton K. Chen Y. (2018). Recovery of over-ground walking after chronic motor complete spinal cord injury. *N. Engl. J. Med.* 379 1244–1250. 10.1056/NEJMoa1803588 30247091

[B2] Angeli C. A. Edgerton V. R. Gerasimenko Y. P. Harkema S. J. (2014). Altering spinal cord excitability enables voluntary movements after chronic complete paralysis in humans. *Brain* 137 1394–1409. 10.1093/brain/awu038 24713270 PMC3999714

[B3] Aslan S. C. Legg Ditterline B. E. Park M. C. Angeli C. A. Rejc E. Chen Y. (2018). Epidural spinal cord stimulation of lumbosacral networks modulates arterial blood pressure in individuals with spinal cord injury-induced cardiovascular deficits. *Front Physiol.* 9:565. 10.3389/fphys.2018.00565 29867586 PMC5968099

[B4] Bloom O. Wecht J. M. Legg Ditterline B. E. Wang S. Ovechkin A. V. Angeli C. A. (2020). Prolonged targeted cardiovascular epidural stimulation improves immunological molecular profile: A case report in chronic severe spinal cord injury. *Front. Syst. Neurosci.* 14:571011. 10.3389/fnsys.2020.571011 33177997 PMC7593242

[B5] Boakye M. Ball T. Dietz N. Sharma M. Angeli C. Rejc E. (2023). Spinal cord epidural stimulation for motor and autonomic function recovery after chronic spinal cord injury: A case series and technical note. *Surg. Neurol. Int.* 14:87. 10.25259/SNI_1074_2022 37025529 PMC10070319

[B6] Darrow D. Balser D. Netoff T. I. Krassioukov A. Phillips A. Parr A. (2019). Epidural spinal cord stimulation facilitates immediate restoration of dormant motor and autonomic supraspinal pathways after chronic neurologically complete spinal cord injury. *J. Neurotrauma* 36 2325–2336. 10.1089/neu.2018.6006 30667299 PMC6648195

[B7] Eisdorfer J. T. Smit R. D. Keefe K. M. Lemay M. A. Smith G. M. Spence A. J. (2020). Epidural electrical stimulation: A review of plasticity mechanisms that are hypothesized to underlie enhanced recovery from spinal cord injury with stimulation. *Front. Mol. Neurosci.* 13:163. 10.3389/fnmol.2020.00163 33013317 PMC7497436

[B8] Hachmann J. T. Yousak A. Wallner J. J. Gad P. N. Edgerton V. R. Gorgey A. S. (2021). Epidural spinal cord stimulation as an intervention for motor recovery after motor complete spinal cord injury. *J. Neurophysiol.* 126 1843–1859. 10.1152/jn.00020.2021 34669485

[B9] Herrity A. N. Aslan S. C. Mesbah S. Siu R. Kalvakuri K. Ugiliweneza B. (2022). Targeting bladder function with network-specific epidural stimulation after chronic spinal cord injury. *Sci. Rep.* 12:11179. 10.1038/s41598-022-15315-2 35778466 PMC9249897

[B10] Herrity A. N. Williams C. S. Angeli C. A. Harkema S. J. Hubscher C. H. (2018). Lumbosacral spinal cord epidural stimulation improves voiding function after human spinal cord injury. *Sci. Rep.* 8:8688. 10.1038/s41598-018-26602-2 29875362 PMC5989228

[B11] Huang X. L. Chen Y. C. Kuo C. C. Tsai S. T. (2022). Potential benefits of spinal cord stimulation treatment on quality of life for paralyzed patients with spinal cord injury. *Tzu Chi. Med. J.* 35 131–136. 10.4103/tcmj.tcmj_102_22 37261296 PMC10227680

[B12] Hwang Y. I. Kim K. S. (2018). Effects of pelvic tilt angles and forced vital capacity in healthy individuals. *J. Phys. Ther. Sci.* 30 82–85. 10.1589/jpts.30.82 29410572 PMC5788781

[B13] Jung Y. Breitbart S. Malvea A. Bhatia A. Ibrahim G. M. Gorodetsky C. (2024). Epidural spinal cord stimulation for spasticity: A systematic review of the literature. *World Neurosurg.* 183 227–238.e5. 10.1016/j.wneu.2023.12.158. 38181878

[B14] Kandhari S. Sharma D. Samuel S. Sharma G. Majumdar P. Edgerton V. R. (2022). Epidural spinal stimulation enables global sensorimotor and autonomic function recovery after complete paralysis: 1^st^ study from India. *IEEE Trans. Neural Syst. Rehabil. Eng.* 30 2052–2059. 10.1109/TNSRE.2022.3158393 35271446

[B15] Kriz J. Nasincova Z. Gallusova V. Vyskocil T. Gregor M. Slaby K. (2024). Muscle excitability scale for the assessment of spastic reflexes in spinal cord injury: Development and evaluation. *Spinal Cord* 62 532–538. 10.1038/s41393-024-01016-2 39020022 PMC11368810

[B16] Legg Ditterline B. E. Wade S. Ugiliweneza B. Singam N. S. Harkema S. J. Stoddard M. F. (2020). Beneficial cardiac structural and functional adaptations after lumbosacral spinal cord epidural stimulation and task-specific interventions: A pilot study. *Front. Neurosci.* 14:554018. 10.3389/fnins.2020.554018 33192245 PMC7643015

[B17] Maurer-Burkhard B. Smoor I. von Reumont A. Deckstein G. Stierle I. Rupp R. (2016). Validity and reliability of a locomotor stage-based functional rating scale in spinal cord injury. *Spinal Cord* 54 619–625. 10.1038/sc.2015.223 26754473

[B18] Mesbah S. Ball T. Angeli C. Rejc E. Dietz N. Ugiliweneza B. (2021). Predictors of volitional motor recovery with epidural stimulation in individuals with chronic spinal cord injury. *Brain* 144 420–433. 10.1093/brain/awaa423 33367527

[B19] Peña Pino I. Hoover C. Venkatesh S. Ahmadi A. Sturtevant D. Patrick N. (2020). Long-term spinal cord stimulation after chronic complete spinal cord injury enables volitional movement in the absence of stimulation. *Front. Syst. Neurosci.* 14:35. 10.3389/fnsys.2020.00035 32714156 PMC7340010

[B20] Rejc E. Angeli C. A. Atkinson D. Harkema S. J. (2017). Motor recovery after activity-based training with spinal cord epidural stimulation in a chronic motor complete paraplegic. *Sci. Rep.* 7:13476. 10.1038/s41598-017-14003-w 29074997 PMC5658385

[B21] Rejc E. Smith A. C. Weber K. A. Ugiliweneza B. Bert R. J. Negahdar M. (2020). Spinal cord imaging markers and recovery of volitional leg movement with spinal cord epidural stimulation in individuals with clinically motor complete spinal cord injury. *Front. Syst. Neurosci.* 14:559313. 10.3389/fnsys.2020.559313 33192348 PMC7654217

[B22] Rosier P. F. W. M. Schaefer W. Lose G. Goldman H. B. Guralnick M. Eustice S. (2017). International continence society good urodynamic practices and terms 2016: Urodynamics, uroflowmetry, cystometry, and pressure-flow study. *Neurourol. Urodyn.* 36 1243–1260. 10.1002/nau.23124 27917521

[B23] Roth E. J. Stenson K. W. Powley S. Oken J. Primack S. Nussbaum S. B. (2010). Expiratory muscle training in spinal cord injury: A randomized controlled trial. *Arch. Phys. Med. Rehabil.* 91 857–861. 10.1016/j.apmr.2010.02.012 20510974

[B24] Rowald A. Komi S. Demesmaeker R. Baaklini E. Hernandez-Charpak S. D. Paoles E. (2022). Activity-dependent spinal cord neuromodulation rapidly restores trunk and leg motor functions after complete paralysis. *Nat. Med.* 28 260–271. 10.1038/s41591-021-01663-5 35132264

[B25] Rybka V. Sediva K. Spackova L. Kolar P. Bradac O. Kriz J. (2024). Epidural spinal cord stimulation can facilitate ejaculatory response in spinal cord injury individuals: A report of two cases. *Int. J. Neurosci.* 134 1357–1364. 10.1080/00207454.2023.2273772 37878354

[B26] Verrills P. Sinclair C. Barnard A. (2016). A review of spinal cord stimulation systems for chronic pain. *J. Pain Res.* 9 481–492. 10.2147/JPR.S108884 27445503 PMC4938148

[B27] Wagner F. B. Mignardot J. B. Le Goff-Mignardot C. G. Demesmaeker R. Komi S. Capogrosso M. (2018). Targeted neurotechnology restores walking in humans with spinal cord injury. *Nature* 563 65–71. 10.1038/s41586-018-0649-2 30382197

[B28] Walter M. Lee A. H. X. Kavanagh A. Phillips A. A. Krassioukov A. V. (2018). Epidural spinal cord stimulation acutely modulates lower urinary tract and bowel function following spinal cord injury: A case report. *Front. Physiol.* 9:1816. 10.3389/fphys.2018.01816 30618826 PMC6305431

[B29] West C. R. Phillips A. A. Squair J. W. Williams A. M. Walter M. Lam T. (2018). Association of epidural stimulation with cardiovascular function in an individual with spinal cord injury. *JAMA Neurol.* 75 630–632. 10.1001/jamaneurol.2017.5055 29459943 PMC5885254

