# Supplementary material for: Effect of epidural spinal cord stimulation in individuals with sensorimotor complete spinal cord injury: a pilot study
Source: Front Syst Neurosci. 2025 Jul 2;19:1622033. doi: 10.3389/fnsys.2025.1622033 (PMC12263693; doi:10.3389/fnsys.2025.1622033)
Supplement: Supplementary file 1 [file Table_1.docx]

Supplementary Material

# Supplementary Table 1 Results for each participant before implantation (M0), at six months (M1), and after one year (M2)

|  |  | Participant A | | | | Participant B | | | | Participant C | | | | Mean | *p*-value* |
| --- | --- | --- | --- | --- | --- | --- | --- | --- | --- | --- | --- | --- | --- | --- | --- |
|  |  | M0 | M1 | M2 | M2-M0 | M0 | M1 | M2 | M2-M0 | M0 | M1 | M2 | M2-M0 | M2-M0 | |
| ADFSCI | AD | 0 | 9 | 1 | 1 | 9 | 2 | 8 | -1 | 56 | 28 | 23 | -33 | -11.0 | 0.423 |
|  | OH | 0 | 3 | 0 | 0 | 8 | 3 | 0 | -8 | 3 | 5 | 1 | -2 | -3.3 | 0.180 |
| NBSS | Incontinence | 9 | 0 | 1 | -8 | 11 | 2 | 0 | -11 | 1 | 3 | 1 | 0 | -6.3 | 0.180 |
|  | Storage and Voiding | 5 | 3 | 4 | -1 | 5 | 7 | 7 | 2 | 9 | 5 | 2 | -7 | -2.0 | 0.593 |
|  | Consequences | 4 | 5 | 6 | 2 | 5 | 4 | 5 | 0 | 5 | 3 | 6 | 1 | 1.0 | 0.180 |
|  | Total | 21 | 10 | 13 | -8 | 24 | 16 | 14 | -10 | 19 | 13 | 11 | -8 | -8.7 | 0.109 |
| NBDS |  | 9 | 8 | 8 | -1 | 11 | 9 | 9 | -2 | 14 | 11 | 9 | -5 | -2.7 | 0.109 |
| IIEF-5 |  | 7 | 13 | 16 | 9 | 20 | 24 | 24 | 4 | 9 | 10 | 10 | 1 | 4.7 | 0.109 |
| SCI-SET | Negative Score | -9 | -3 | -2 | 7 | -14 | -2 | -1 | 13 | -23 | -12 | -7 | 16 | 12.0 | 0.109 |
|  | Positive Score | 0 | 0 | 0 | 0 | 4 | 2 | 1 | -3 | 1 | 0 | 0 | -1 | -1.3 | 0.180 |
|  | Total Score | -9 | -3 | -2 | 7 | 10 | 0 | 0 | 10 | -22 | -12 | -7 | 15 | 4.0 | 0.593 |
| NPS |  | 0 | 0 | 0 | 0 | 9 | 0 | 0 | -9 | 60 | 36 | 36 | -24 | -11.0 | 0.180 |
| WHOQOL-BREF | Physical Health | 27 | 26 | 27 | 0 | 28 | 32 | 29 | 1 | 20 | 22 | 25 | 5 | 2.0 | 0.180 |
|  | Psychological | 24 | 20 | 19 | -5 | 24 | 26 | 29 | 5 | 21 | 22 | 22 | 1 | 0.3 | 0.789 |
|  | Social Relationships | 11 | 11 | 12 | 1 | 14 | 15 | 14 | 0 | 11 | 11 | 11 | 0 | 0.3 | ** |
|  | Environment | 30 | 30 | 31 | 1 | 32 | 36 | 34 | 2 | 31 | 30 | 33 | 2 | 1.7 | 0.109 |
|  | Mean | 23 | 21.8 | 22.3 | -0.7 | 24.5 | 27.3 | 26.5 | 2 | 20.8 | 21.3 | 22.8 | 2 | 1.1 | 0.285 |
| ISNCSCI | NLI | T2 | T2 | T3 |  | T4 | T3 | T4 |  | T4 | T4 | T4 |  |  |  |
|  | AIS | A | A | A |  | A | A | A |  | A | A | A |  |  |  |
|  | SSLT right | 19 | 21 | 20 | 1 | 23 | 23 | 24 | 1 | 22 | 23 | 22 | 0 | 0.7 | 0.180 |
|  | SSLT left | 19 | 19 | 21 | 2 | 23 | 24 | 24 | 1 | 23 | 23 | 23 | 0 | 1.0 | 0.180 |
|  | SSPP right | 18 | 21 | 21 | 3 | 22 | 23 | 23 | 1 | 22 | 22 | 22 | 0 | 1.3 | 0.180 |
|  | SSPP left | 20 | 21 | 21 | 1 | 22 | 22 | 22 | 0 | 22 | 22 | 22 | 0 | 0.3 | ** |
| Spasticity | MES R | 1 |  | 1 | 0 | 4 | 1 | 3 | -1 | 0 | 1 | 1 | 1 | 0.0 | 1.000 |
|  | MES L | 1 |  | 1 | 0 | 4 | 1 | 2 | -2 | 0 | 0 | 1 | 1 | -0.3 | 0.655 |
|  | MAS QF R | 1.5 |  | 1 | -0.5 | 2 | 3 | 3 | 1 | 0 | 0 | 0 | 0 | 0.2 | 0.655 |
|  | MAS QF L | 1 |  | 1 | 0 | 3 | 1.5 | 1 | -2 | 0 | 0 | 0 | 0 | -0.7 | ** |
|  | MAS BF R | 1 |  | 1 | 0 | 3 | 2 | 3 | 0 | 0 | 0 | 0 | 0 | 0.0 | ** |
|  | MAS BF L | 0 |  | 0 | 0 | 2 | 2 | 1 | -1 | 0 | 0 | 0 | 0 | -0.3 | ** |
|  | MAS TS R | 1.5 |  | 0 | -1.5 | 1 | 2 | 1.5 | 0.5 | 0 | 0 | 0 | 0 | -0.3 | 0.655 |
|  | MAS TS L | 0 |  | 0 | 0 | 1 | 0 | 1 | 0 | 0 | 0 | 0 | 0 | 0.0 | ** |
| Spirometry | FVC (L) | 5.7 | 5.9 | 5.5 | -0.2 | 4.4 | 4.3 | 4.0 | -0.4 | 4.7 | 4.0 | 4.0 | -0.7 | -0.4 | 0.109 |
|  | FEV1 (L) | 4.0 | 4.2 | 4.3 | 0.3 | 3.9 | 4.0 | 3.7 | -0.2 | 4.5 | 3.9 | 3.8 | -0.6 | -0.2 | 0.593 |
|  | PEF (L/s) | 7.9 | 8.4 | 8.8 | 0.9 | 8.6 | 7.5 | 8.8 | 0.1 | 6.9 | 7.9 | 12.5 | 5.6 | 2.2 | 0.109 |
|  | FEV1/FVC (%) | 69.4 | 71.8 | 75.9 | 6.5 | 88.7 | 92.8 | 93.0 | 4.3 | 95.9 | 98 | 96.0 | 0.1 | 3.6 | 0.109 |
|  | MIP (cmH_2_O) | 78.0 | 82.0 | 97.0 | 19.0 | 94.0 | 52.0 | 87.0 | -7.0 | 97.0 | 128 | 153 | 56.0 | 22.7 | 0.285 |
|  | MEP (cmH_2_O) | 91.0 | 55.0 | 90.0 | -1.0 | 62.0 | 77.0 | 87.0 | 25.0 | 74.0 | 91.0 | 92.0 | 18.0 | 14.0 | 0.285 |
| TST | Static | 6 | 6 | 6 | 0 | 6 | 6 | 6 | 0 | 6 | 6 | 6 | 0 | 0.0 | ** |
|  | Dynamic | 4 | 4 | 4 | 0 | 4 | 4 | 4 | 0 | 4 | 4 | 4 | 0 | 0.0 | ** |
|  | Dynamic with UE | 10 | 10 | 10 | 0 | 8 | 10 | 10 | 2 | 6 | 6 | 6 | 0 | 0.7 | ** |
|  | Total | 20 | 20 | 20 | 0 | 18 | 20 | 20 | 2 | 16 | 16 | 16 | 0 | 0.7 | ** |
| HUTT | SBP ∆ | -9.6 | -22.4 | -40.1 | -30.5 | -45.2 | -28.5 | -30.2 | 15.0 | 1.4 | 8.0 | 6.0 | 4.6 | -3.7 | 1.000 |
|  | DBP ∆ | 0.7 | -13.4 | -17.9 | -18.6 | -20.0 | -4.9 | -14.2 | 5.8 | 11.4 | 13.4 | 12.0 | 0.6 | -4.0 | 1.000 |
|  | HR ∆ | 30.1 | 30.1 | 54.6 | 24.5 | 51.6 | 44.7 | 39.5 | -12.1 | 29.0 | 25.0 | 23.8 | -5.2 | 2.4 | 1.000 |

ADFSCI, Autonomic Dysfunction Following Spinal Cord Injury; AD, Autonomic Dysreflexia; OH, Orthostatic Hypotension; NBSS, Neurogenic Bladder Symptom Score; NBDS, Neurogenic Bowel Dysfunction Score; IIEF-5, International Index of Erectile Function; SCI-SET, Spinal Cord Injury Spasticity Evaluation Tool; NPS, Neuropathic Pain Scale; WHOQOL-BREF, World Health Organisation Quality Of Life – abbreviated version; ISNCSCI, International Standards for Neurological Classification of Spinal Cord Injury; NLI, Neurological Level of Injury; AIS, American Spinal Injury Association Impairment Scale; LTSS, Light Touch Sensory Subscore; PPSS, Pin Prick Sensory Subscore; MES, Muscle Excitability Scale; MAS, Modified Ashworth Scale; QF, Quadriceps Femoris; BF, Biceps Femoris; TS, Triceps Surae; FVC, Forced Vital Capacity; FEV1, Forced Vital Capacity in 1 second; MIP, Maximum Inspiratory Pressure; MEP, Maximum Expiratory Pressure; TST, Trunk Stability Test; UE, Upper Extremities; HUTT, Head-Up Tilt Test; SBP ∆, Difference in Systolic Blood Pressure between 0 and 60 degrees; DBP ∆, Difference in Diastolic Blood Pressure between 0 and 60 degrees; HR ∆, Difference in Heart Rate between 0 and 60 degrees

*Wilcoxon Matched Pair Test

** *p*-value is missing due to insufficient degrees of freedom in the Wilcoxon test
