# Supplementary material for: Effect of epidural spinal cord stimulation in individuals with sensorimotor complete spinal cord injury: a pilot study
Source: Front Syst Neurosci. 2025 Jul 2;19:1622033. doi: 10.3389/fnsys.2025.1622033 (PMC12263693; doi:10.3389/fnsys.2025.1622033)
Supplement: Supplementary file 3 [file Data_Sheet_1.docx]

Supplementary Material

# Supplementary Method Functional Magnetic Resonance Imaging: Data Acquisition and Analysis

FMR data were acquired on a Philips dStream 1.5 T machine using a 16-channel head coil. The echo planar imaging (EPI) measurement sequence had the following parameters: TR 3000 ms, TE 50 ms, voxel size 3x3x3 mm, FOV 208x208 mm, and 100 epochs measured in total. A standard block scheme of the motor paradigm was used, alternating the activation time of the left or right limb with the rest period after regular intervals. The length of one block of the paradigm was 5 epochs (15 s).

The results were analyzed with a general linear model using the SPM12 toolbox in the Mathworks Matlab 2023b program. A standard procedure for single-subject processing was followed, which included segmentation, normalization, and smoothing with a 6 mm Gaussian kernel. No specific evaluation tool was employed to compare the results.
